# Effectiveness of multicomponent treatment in patients with fibromyalgia: protocol for a systematic review and meta-analysis

**DOI:** 10.1186/s13643-022-01944-1

**Published:** 2022-04-15

**Authors:** Felipe Araya-Quintanilla, Héctor Gutiérrez-Espinoza, Jorge Fuentes, Fernanda Prieto-Lafrentz, Leonardo Pavez, Carlos Cristi-Montero, Iván Cavero-Redondo, Celia Álvarez-Bueno

**Affiliations:** 1 Rehabilitation in Health Research Center (CIRES), Universidad de Las Américas, Manuel Montt Avenue 948, 7510549 Santiago, Chile; 2 Exercise and Rehabilitation Sciences Laboratory, School of Physical Therapy, Faculty of Rehabilitation Sciences, Universidad Andres Bello, Santiago, Chile; 3 Department of Physical Therapy, Catholic University of Maule, Talca, Chile; 4 Department of Physical Therapy, University of Alberta, Edmonton, Alberta Canada; 5 Faculty of Economy and Business, Universidad Andres Bello, Santiago, Chile; 6 Facultad de Medicina Veterinaria y Agronomía, Universidad de las Américas, Santiago, Chile; 7 Departamento de Ciencias Químicas y Biológicas, Universidad Bernardo O’Higgins, Santiago, Chile; 8 IRyS Group, Physical Education School, Pontificia Universidad Católica de Valparaíso, Viña del Mar, Chile; 9 Universidad de Castilla-La Mancha, Health and Social Research Center, Cuenca, Spain; 10 Universidad Politécnica y Artística del Paraguay, Asunción, Paraguay

**Keywords:** Fibromyalgia, Multicomponent treatment, Pain, Systematic review, Meta-analysis, Protocol

## Abstract

**Background:**

The purpose of this protocol is to provide a new systematic review with meta-analysis using the current methodology to compare the effectiveness of multicomponent treatment versus other interventions for patients with fibromyalgia.

**Methods:**

This protocol conforms to the Preferred Reporting Items for Systematic Review and Meta-Analysis Protocols (PRISMA-P) and the recommendations of the Cochrane Collaboration Handbook. An electronic search will be conducted in MEDLINE, EMBASE, Web of Science, Cochrane CENTRAL, LILACS, CINAHL, and PEDro, from inception until April 2022. There will be no language restrictions. The Cochrane Collaboration tool for assessing the risk of bias (RoB2) will be used. The Grading of Recommendations, Assessment, Development and Evaluation (GRADE) scale will be used to evaluate the strength of the evidence. The Hartung-Knapp-Sidik-Jonkman random effects or Mantel-Haenszel fixed effects methods will be used, depending on the heterogeneity, to compute a pooled estimate of the mean difference (MD) or standardized mean difference (SMD) and respective 95% confidence intervals for clinical outcomes.

**Discussion:**

This systematic review will synthesize evidence on the effectiveness of multicomponent treatment in patients with fibromyalgia and could add important evidence in the treatment of FM to improve clinical practice and decision-making/actions in this field. This new systematic review will try to show the effects of multicomponent treatment by type (endurance, resistance, stretching, or mind-body exercises [pilates or taichi]) and intensity (light, moderate, moderate-to-vigorous, vigorous) of exercise in patients with FM. The results will be disseminated by publication in a peer-reviewed journal. Ethics approval will not be needed because the data used for this systematic review will be obtained from individual trials and there will be no concerns about privacy. However, if we identify ethical issues during the development of the systematic review, these findings will be reported in the discussion of the study.

**Systematic review registration:**

PROSPERO CRD42020142082.

## Strengths and limitations

- This systematic review could add important evidence in the treatment of FM to improve clinical practice and decision-making.
- This review provides the evidence assessed with the GRADE system to rate the quality of evidence of multicomponent treatment in FM.
- Different intensities and types of multicomponent intervention could be a source of different results and heterogeneity between studies and may limit the quality of evidence from this meta-analysis and systematic review.
- We will search seven databases and manual references; however, we could miss clinical trials relevant to our research.

## Background

According to the evidence, fibromyalgia (FM) is a chronic disease that includes musculoskeletal pain, fatigue, and cognitive problems [1, 2]. The prevalence of FM in the general population ranges from 2 to 6.6% worldwide, with higher frequency among women [3, 4]. This clinical condition can affect patients’ quality of life and cause high healthcare cost, with patients often needing cost-effective treatment options [5–7]. Currently, different evidence-based approaches have been published to provide patients and physicians with multidisciplinary treatment options for FM [8, 9]. Previous systematic reviews have shown that pharmacological treatments, including pregabalin, amitriptyline, and milnacipran, are controversial and produce only moderate effect in patients with FM [10–13]. Moreover, other studies indicate that multidisciplinary interventions, such as multicomponent treatment, have positive results on FM symptoms in the short term for pain intensity, fatigue, depressive symptoms, and physical function [14, 15], specifically when including exercise and cognitive behavioral therapy [16–18]. Multicomponent treatment is defined as an intervention that involves a combination of aerobic exercise, cognitive behavioral therapy, and/or education [16]. However, the multifactorial nature of FM, its wide variety of symptoms, and different therapeutic interventions included in multicomponent treatment could interfere with treatment success [14, 19]. A recent overview of the guidelines [20, 21] has shown inconsistent results for multicomponent treatments in the management of FM. Thus, it is difficult to establish which type (endurance, resistance, stretching, or mind-body exercises [pilates or taichi]) and intensity (light, moderate, moderate-to-vigorous, vigorous) of physical exercise or specific multicomponent treatment therapy combination is clinically more useful for patients with FM. The recently updated guidelines of the European League Against Rheumatism and the National Institute for Health and Care Excellence [22–24] concluded that the recommendation for multidisciplinary interventions is strong and has a “low to moderate effect” on pain relief and fatigue improvement in FM. Subsequently, they suggest a multidisciplinary approach for patients with chronic pain. However, it has been suggested that further studies should be conducted with a clearer methodology to optimize results in patients with FM. Additionally, the results of previous clinical trials into the effect of multicomponent treatment on the different symptoms of FM have been inconsistent; therefore, it is necessary for a systematic review to present a clear and transparent procedure for systematically reviewing, evaluating, and summarizing existing evidence [21, 25]. Other systematic reviews have been published [10, 26]: one [10] shows the effects of pharmacological interventions in FM and another [26] shows different non-pharmacological interventions in chronic pain populations but not specifically FM. Furthermore, multicomponent treatment has not been studied on other important clinical outcomes such as kinesiophobia, catastrophizing, level of anxiety, stress and depression, and quality of sleep. In fact, there has been no systematic review that includes a current methodology and new trials to study the effectiveness of multicomponent treatment in the medium and long term for patients with FM. Therefore, the aim of this systematic review protocol study will be to establish a current methodology to perform a systematic review and meta-analysis to determine the effectiveness of multicomponent treatment compared to other interventions in patients with FM.

### Objective

This protocol study aims to provide a standardized and clear procedure for a systematic review and meta-analysis aimed at synthesizing all the available evidence about the effectiveness of multicomponent treatment by type (endurance, resistance, stretching, or mind-body exercises [pilates or taichi]) and intensity (light, moderate, moderate-to-vigorous, vigorous) compared to other interventions, such as pharmacological treatment, drug therapy, and other different types of physical exercise, on physical function, pain catastrophizing, kinesiophobia, quality of life, sleep quality, and level of depression and anxiety in patients with FM.

## Methods and analysis

This systematic review protocol has been registered in the PROSPERO database (registration number: CRD42020142082). It will be conducted according to the guidelines of the Preferred Reporting Items for Systematic Review and Meta-Analysis Protocols (PRISMA-P) [27] and the Cochrane Handbook for Systematic Reviews of Interventions [28]. Financial support for the investigation was provided by Universidad de las Americas funding.

### Inclusion and exclusion criteria

The study will include research defined by the following characteristics: type of study (randomized clinical trials); type of participants (subjects older than 18 years of age with a medical diagnosis of FM based on the American College of Rheumatology) [29]; type of intervention; multicomponent treatment, including any type (endurance, resistance, stretching, or mind-body exercises [pilates or taichi]) of physical exercise, cognitive behavioral therapy, and/or education; and type of comparison, including other interventions such as pharmacological treatment, wait and see, other different types of physical exercise (e.g., aerobic, stretching, or strength exercise) and complementary therapy. Finally, this review will include studies in which the outcome of interest is pain intensity, physical function, pain catastrophizing, kinesiophobia, quality of life, sleep quality, and level of depression. We will exclude studies with the following characteristics: studies reporting pre-post analysis without a comparison group; studies involving subjects with other pathologies and conditions, such as chronic fatigue syndrome, myalgic encephalomyelitis, and chronic cancer pain; studies using scales or diagnostic criteria other than those proposed by the American College of Rheumatology; and studies involving subjects with metabolic disorder and/or uncontrolled comorbidities.

### Main outcomes

The primary outcome was pain intensity, measured with the Visual Analogue Scale, the Numeric Rating Scale, or other scales. Physical function and health status were measured with the Fibromyalgia Inventory Questionnaire or other questionnaires. Secondary outcomes were pain catastrophizing (e.g., Pain Catastrophizing Scale), kinesiophobia (e.g., Tampa Scale of Kinesiophobia), quality of life (e.g., SF-36, QoL-16), sleep quality (e.g., Pittsburgh Scale), level of fatigue (e.g., Fatigue Assessment Scale), level of depression and anxiety (e.g., Hospital Anxiety and Depression Scale), and level of stress (e.g., Perceived Stress Scale 4).

### Search strategy

Relevant studies of multicomponent treatment for FM will be obtained through an extensive computerized search from the following bibliographic databases: MEDLINE (via PubMed), EMBASE, Web of Science, Cochrane Central Register of Controlled Trials (CENTRAL), Latin American and the Caribbean Literature in Health Sciences (LILACS), Cumulative Index to Nursing and Allied Health Literature (CINAHL), and Physiotherapy Evidence Database (PEDro), from inception until April 2022. The literature search procedure will be complemented by manually searching the references of the identified articles to detect additional studies of interest. Also, we will include InterTASC Information Specialists’ Sub-Group (ISSG) Search Filter Resource proposed by the Cochrane collaboration to perform the most sensitive database searches. Combinations of the following keywords will be used in the search: “fibromyalgia,” “chronic fatigue syndrome,” “diffuse myofascial pain syndrome,” “multicomponent treatment,” “multimodal therapy,” “multidisciplinary approach,” “physical exercise,” “exercise therapy,” “randomized clinical trial,” and “controlled clinical trial” (see Table 1).

**Table 1:** Search strategy

Number	Search terms
1	Fibromyalgia
2	Chronic fatigue syndrome
3	Diffuse Myofascial Pain Syndrome
4	#1 OR #2 OR #3
5	Multicomponent treatment
6	Multimodal therapy
7	Multidisciplinary approach
8	Physical exercise
9	Exercise Therapy
10	#5 OR #6 OR #7 OR #8 OR #9
11	#4 AND #10
12	Randomized clinical trial
13	Randomized controlled trial
14	#12 OR #13
15	Humans
16	Animals
17	#15 NOT # 16
18	#11 AND #14 AND #17

### Selection and analysis of trials

**Fig. 1:**
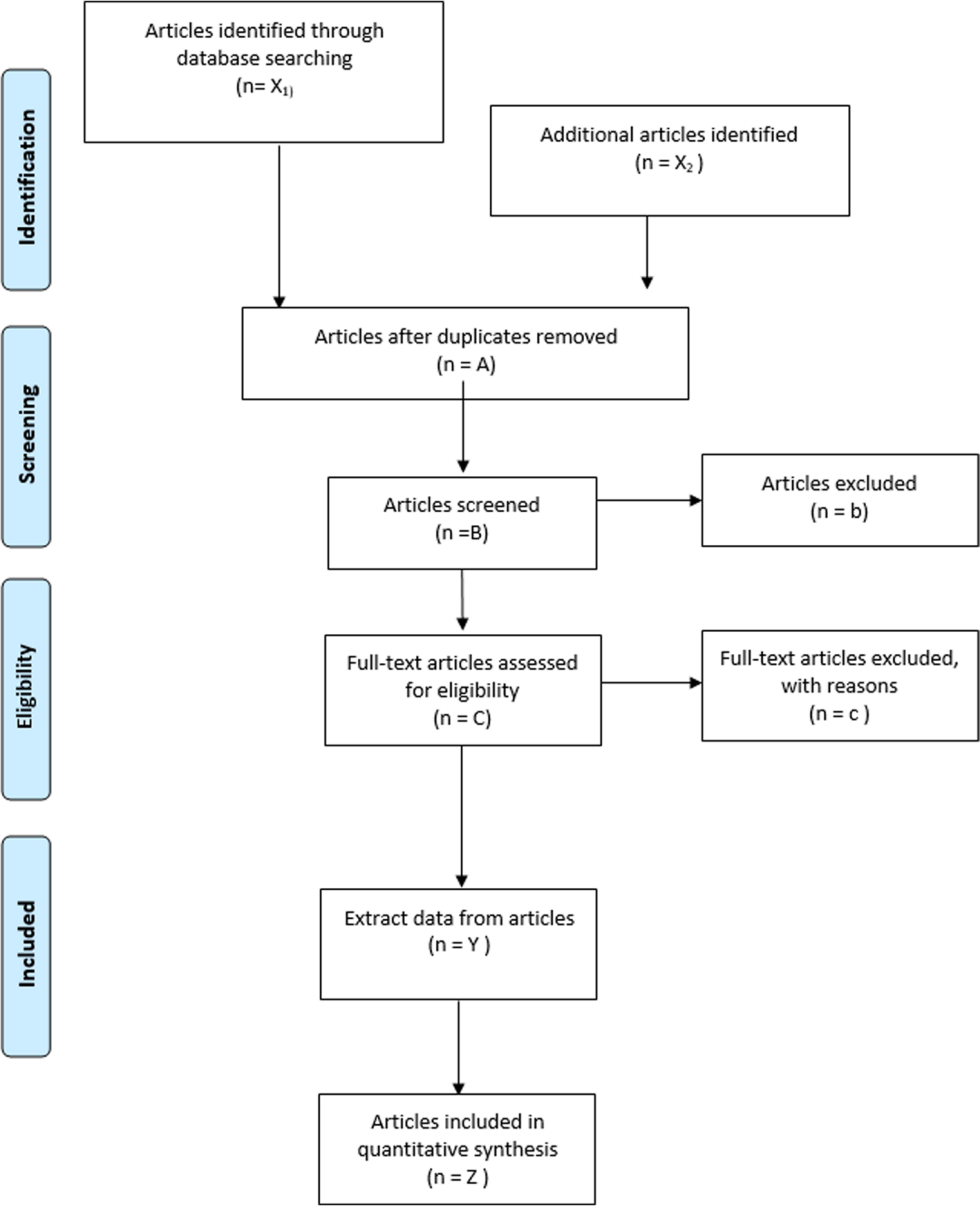
PRISMA flow diagram of identification, screening, eligibility, and inclusion of studies

After the search is performed, two reviewers will independently screen the titles and retrieve the abstracts. The full text of manuscripts selected for inclusion will be examined and the inclusion and exclusion criteria will be applied (Fig. 1). The reviewers will not be blinded to authors, institutions, or journals. Disagreements between reviewers will be solved by consensus or through the participation of a third reviewer. The reviewers will independently extract the following information from the included studies: author and year of publication, design of the trial, country, type of intervention (multicomponent treatment and other interventions, multicomponent treatment include any different type of exercise such as endurance, resistance, stretching, or mind-body exercises [pilates or taichi]), intervention characteristics (intensity [light, moderate, moderate-to-vigorous, vigorous], length, and setting), population characteristics, number of participants, age of participants, outcomes studied, and results. In addition, the clinical significance/relevance of included studies will be reported by effect size and/or minimum clinically important difference (see Table 2). Any disagreement between reviewers will be resolved by consensus. Finally, study authors will be asked to supply any missing data.

**Table 2:** Characteristics of the trials included in the systematic review and meta-analyses

Reference	Design	Country	Population	Intervention	Control	Results/follow-up
Authors/years	RCTs, CCTs	Countries where the trial was conducted	Patients with fibromyalgia	Type, dose, and characteristics	Type, dose, and characteristics	ΔX: SD: *P* value

*RCTs* randomized clinical trial, *CTTs* controlled trial, *x* mean, *SD* standard deviation, *Δx* mean difference

### Evaluation of the risk of bias (RoB2)

Two reviewers will independently assess the risk of bias according to the Cochrane Collaboration Handbook recommendations [28]. Disagreements will be solved by consensus or through the participation of a third reviewer. The randomized clinical trials will be assessed using the Cochrane Collaboration tool for assessing the risk of bias (RoB2) [30]. This tool assesses the risk of bias according to six domains: bias arising from the randomization process, bias due to deviations from intended interventions, bias due to missing outcome data; bias in the measurement of outcome, bias in the selection of the reported result, and overall bias. Overall bias will be considered as “low risk of bias” if the paper has been classified as low risk in all domains, “some concerns” if there is at least one domain with a rating of some concern, and “high risk of bias” if there is at least one domain with a high risk or several domains with some concerns that could affect the validity of the results. The agreement rate between reviewers will be calculated using kappa statistics.

### Grading the quality of evidence

The Grading of Recommendations, Assessment, Development and Evaluation (GRADE) tool will be used to assess the quality of the evidence and make recommendations [31, 32]. Each outcome could obtain a high, moderate, low, or very low evidence value depending on the study design, risk of bias, inconsistency, indirect evidence, imprecision, and publication bias.

### Data analysis

Descriptive analyses will be conducted for those studies that present insufficient data for overall pooling, and narrative synthesis will be performed following the Cochrane Collaboration guidelines [28]. The Hartung-Knapp-Sidik-Jonkman random effects (*I*^*2*^ ≥ 55%) or Mantel-Haenszel fixed effects method in the case of unimportant statistical inconsistency between studies (*I*^*2*^ ≤ 55%) to produce more conservative confidence intervals (CIs) will be used [33]. In addition, visual inspection was considered for overlap with the CI. Meta-analyses will be performed using the pooled estimate of the mean difference (MD) or standardized mean difference (SMD) for outcomes measured with different scales and respective 95% CIs. The inconsistency of results across studies will be evaluated using the *I*^*2*^ statistic, which will be considered as “might not be important” (0–40%), “may represent moderate” (30–60%), “may represent substantial” (50–90%), and “considerable” (75–100%) heterogeneity [34]. The corresponding *P* values also will be considered. Publication bias will be evaluated through a visual inspection of funnel plots, as well as by using the method proposed by Egger [35]. The meta-analysis will be performed using the RevMan 5.4 program. The synthesis and quality of evidence for each outcome will be assessed by GRADE profiling (GRADEpro) to import the data from Review Manager 5.4 (RevMan 5.4) in order to create a “summary of findings” table. This approach entails the downgrading of evidence from high to moderate to low and very low quality based on certain criteria: (1) for study limitation if the majority of studies (> 50%) were rated as high risk of bias; (2) for inconsistency if heterogeneity was greater than the accepted low level (*I*^*2*^ > 40%); (3) for indirectness if the multicomponent treatment session does not correspond to what is used in clinical practice; and (4) for imprecision if meta-analysis had a small sample size (*n* < 300). When necessary, authors of potentially eligible studies will be contacted to obtain missing data.

### Missing data imputation

Following the Cochrane Handbook for Systematic Reviews of Interventions [28], when there is not enough information in the studies to calculate standard deviations (SDs), they were imputed using standard errors (SE), confidence intervals (CIs), *t*-statistic, or *P* values, using the following formulas: (i) SD = sqrt (sample size) * SE; (ii) SD = {(upper limit CI − lower limit CI)/3.92} * sqrt (sample size); (iii) SD = (mean difference/*t*-statistic) * sqrt (sample size); (iv) or SD = {−0.862 þ sqrt [0.743–2.404 * log (*P* value)]} * sqrt (sample size), respectively. When sample size was not provided in the analysis table, the sample size was extracted from the descriptive statistics.

### Analysis by subgroups

If possible, subgroup analysis will be performed based on the type of intervention, as this is a characteristic that can modify the results for the different outcomes, including the type of multicomponent treatment and comparators such as physical exercise, standard interventions, and drug therapy. Additionally, if possible, subgroup analyses will be based on the intensity of exercise performed in the multicomponent treatment group. Finally, to assess the robustness of the summary estimates and detect whether any particular study explains a large proportion of the heterogeneity, sensitivity analyses will be performed, removing the included studies one-by-one from the pooled analyses.

### Ethics and dissemination

This systematic review will synthesize the evidence on the effectiveness of multicomponent treatment in patients with FM. The results will be disseminated by publication in a peer-reviewed journal. Ethics approval will not be needed because the data used for this systematic review will be obtained from individual trials contributing primary data for meta-analyses. There will be no concerns about the privacy of patients because all data will be fully anonymized prior to being imported into our database. However, if we identify ethical problems during development of the systematic review, these findings will be reported in the discussion of the study.

## Discussion

FM is one of the most common musculoskeletal disorders of unknown cause involving adults, especially women [36]. A multidisciplinary approach is recommended for the treatment of FM [21, 22, 24]. Although pharmacotherapy is prescribed as a first-line treatment for FM, its efficacy remains controversial [10–13, 37]. Recent clinical trials comparing multicomponent treatment versus pharmacotherapy and other interventions in patients with FM have been published but the inconsistent results have made it difficult to draw conclusions from the newly available evidence [15, 38–43]. This protocol aims to provide a new synthesis that overcomes the limitations existing in previous systematic reviews and meta-analyses, which only assess the effect of multicomponent treatment on FM without considering the type of exercise or the intensity of the intervention used [9, 10, 14, 20]. To our knowledge, this will be the first systematic review conducted and reported according to the current highest methodological standard to identify methodological and clinical aspects to be considered for decision-making by medical and physiotherapist professionals in the healthcare of FM patients. Therefore, it is beneficial to perform a systematic review and meta-analysis, including new clinical trials, to determine the magnitude of change in the main FM symptoms in order to improve the estimations and rate the quality of evidence using GRADE for systematic review.

Pharmacotherapy remains the most common treatment to manage the FM condition. Thus, due to the low compliance of patients to recommendations for physical activity or a healthy lifestyle, physicians trend to medicalize the disease [44]. However, the 50% of patients with FM do not improve significantly with pharmacological treatment [41, 45].

Three significant concerns exist in this field that should be considered. First, there are several mixed clinical trials where the effects of physical exercise with education and other interventions (e.g., cognitive behavioral therapy, medical education, stretching exercise) could be a source of heterogeneity. Second, we may be able to find studies where unsupervised exercise is prescribed. No special consideration will be made in the analysis and only the type and intensity of the prescribed exercise or other interventions will be taken into account. However, the lack of direct supervision could threaten the validity of the data. Third, the controlled clinical trial can be affected by selection bias and allocation concealment, so the homogeneity of the basal characteristics of the intervention and control groups is not ensured.

Potential limitations are those common to the systematic reviews, which are (1) bias due to publication and information of clinical trials; (2) although we will search seven databases and include a manual references search, we could miss articles relevant to our research; (3) it is possible to have a high degree of clinical and statistical heterogeneity among the included studies, with potential sources of heterogeneity being different intensities and types of intervention and different scales used to measure the outcome; and (4) the analyses, reporting methods, and findings of the included studies could be a source of bias in grading the quality of evidence. This systematic review could add important evidence in the treatment of FM to improve clinical practice and decision-making/actions in this field. The novel statistical analysis will try to show the effects of multicomponent treatment by type and intensity of exercise in patients with FM. This approach and the quality of the evidence assessed with the GRADE system will provide the strongest evidence to date on the effect of multicomponent treatment of FM symptoms.

## Data Availability

Not applicable.

## Abbreviations

CI: Confidence interval
FM: Fibromyalgia
MD: Mean difference
PRISMA-P: Preferred Reporting Items for Systematic Review and Meta-Analysis Protocols
RoB: Risk of bias
SMD: Standard mean difference
